# Oxygenation correlates with lung aeration during unsupported spontaneous breathing in porcine lung collapse model

**DOI:** 10.1186/cc10728

**Published:** 2012-03-20

**Authors:** L Vimlati, A Larsson, G Hedenstierna, M Lichtwarck-Aschoff

**Affiliations:** 1 Uppsala University, Uppsala, Sweden

## Introduction

We investigated whether oxygenation correlates with lung aeration during unsupported spontaneous breathing (SB) and mechanical ventilation (MV) in a porcine lung collapse model.

## Methods

In 14 anesthetized supine piglets, lung collapse was induced by negative pressure application (NPA) to the endotracheal tube. Eight animals resumed SB 5 minutes after NPA, six animals were kept on MV at a respiratory rate and tidal volume corresponding to SB. Thoracic CTs and arterial blood gases were taken 2.5 and 30 minutes after NPA. Spearman rank correlation was used for testing; values are given as mean (95% CI).

## Results

**Figure 1:**
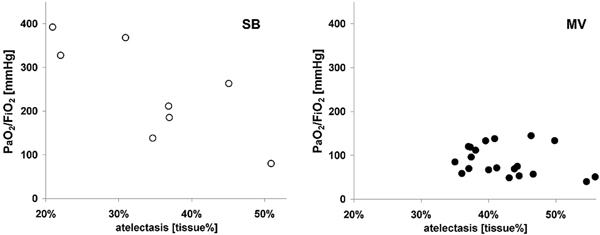
**PaO_2_/FiO_2 _plotted against the proportion of atelectatic lung tissue**. Open circles, SB; solid circles, MV.

Thirty minutes after NPA the amount of lung tissue in collapsed regions was similar in both groups (MV: 40% (36 to 44), SB: 35% (26 to 43); *P *= 0.22). Resuming SB, PaO_2_/FiO_2 _improved significantly more with less amount of collapsed lung tissue 2.5 minutes after NPA (*r *= -0.87, *P *= 0.033). During SB a significant negative correlation between PaO_2_/FiO_2 _and the amount of collapsed lung tissue (*r *= -0.76, *P *= 0.038) was observed; no such correlation could be seen during MV (*r *= -0.3, *P *= 0.2) (Figure 1).

## Conclusion

In porcine lung collapse PaO_2_/FiO_2 _correlates with lung aeration during unsupported SB, but not during MV at a similar breathing pattern. The less lung collapse the animals have, the more PaO_2_/FiO_2 _improves resuming SB.
